# Molecular Dissection of Mesenchymal–Epithelial Interactions in the Hair Follicle

**DOI:** 10.1371/journal.pbio.0030331

**Published:** 2005-09-20

**Authors:** Michael Rendl, Lisa Lewis, Elaine Fuchs

**Affiliations:** **1**Howard Hughes Medical Institute, The Rockefeller University, New York, New York, United States of America; Duke University Medical CenterUnited States of America

## Abstract

De novo hair follicle formation in embryonic skin and new hair growth in adult skin are initiated when specialized mesenchymal dermal papilla (DP) cells send cues to multipotent epithelial stem cells. Subsequently, DP cells are enveloped by epithelial stem cell progeny and other cell types to form a niche orchestrating hair growth. Understanding the general biological principles that govern the mesenchymal–epithelial interactions within the DP niche, however, has been hampered so far by the lack of systematic approaches to dissect the complete molecular make-up of this complex tissue. Here, we take a novel multicolor labeling approach, using cell type–specific transgenic expression of red and green fluorescent proteins in combination with immunolabeling of specific antigens, to isolate pure populations of DP and four of its surrounding cell types: dermal fibroblasts, melanocytes, and two different populations of epithelial progenitors (matrix and outer root sheath cells). By defining their transcriptional profiles, we develop molecular signatures characteristic for the DP and its niche. Validating the functional importance of these signatures is a group of genes linked to hair disorders that have been largely unexplored. Additionally, the DP signature reveals novel signaling and transcription regulators that distinguish them from other cell types. The mesenchymal–epithelial signatures include key factors previously implicated in ectodermal-neural fate determination, as well as a myriad of regulators of bone morphogenetic protein signaling. These findings establish a foundation for future functional analyses of the roles of these genes in hair development. Overall, our strategy illustrates how knowledge of the genes uniquely expressed by each cell type residing in a complex niche can reveal important new insights into the biology of the tissue and its associated disease states.

## Abstract 2

Determining the molecular signature of the cells that orchestrate hair follicle growth generates new insights that will aid in understanding the normal biology and disease states of this tissue.

## Introduction

**Figure 1:**
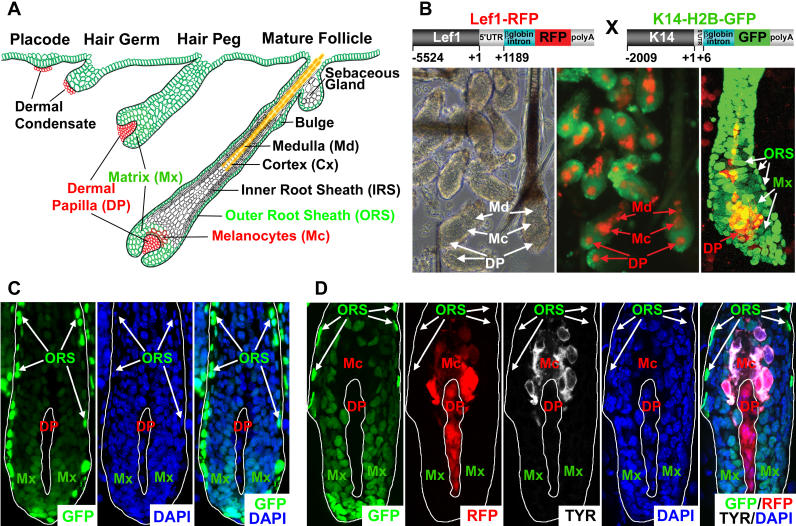
Hair Follicle Morphogenesis and Differential Expression of Lef1-RFP and K14-H2BGFP in All Cells of the Mature Follicle (A) Schematic depicts follicle morphogenesis, which begins in waves in mouse backskin at approximately embryonic day 15 (~E15) and is complete at approximately postnatal day 4 (~P4). (B) Transgene constructs and mice. The transgenes used for injection are shown in the stick diagrams. Nucleotide residues encompassing the gene fragments cloned are noted (+1 is the transcription initiation site). For each transgene, three transgenic lines were engineered; all were phenotypically normal. Data shown are from P4 backskin follicles of mice harboring both transgenes. Left and middle images are phase contrast and epifluorescence images, respectively, of hair follicles after dispase and collagenase treatment. Right image shows 3D reconstruction of a confocal Z-stack of a follicle showing that most of the RFP resides in the center of the follicle bulb. DP, dermal papilla; Mc, melanocytes; Md, medulla; Mx, matrix; ORS, outer root sheath. (C) Section of K14-H2BGFP follicle, which at P4 (shown) is fully mature. Images shown are DAPI and epifluorescence channels, separately, and merged. Note the approximately 3-fold higher levels of GFP in ORS nuclei (arrows) compared to Mx nuclei and its progeny. White lines demarcate the mesenchymal–epithelial boundaries, which are separated by a basement membrane of ECM. (D) Four-color confocal image of a section of a K14-H2BGFP and Lef1-RFP double transgenic follicle at P4. In addition to H2BGFP (green) and RFP (red) epifluorescence, the follicle was labeled with DAPI (blue) and Abs against tyrosinase (secondary Abs are against Cy5 in the far-red and white was used as a pseudocolor). Note that the anti-tyrosinase antibody labels the RFP-positive Mc and demarcates them from the RFP-positive, anti- tyrosinase negative DP. Note also that Mc are located on the epithelial side of the basement membrane, denoted by the white lines.

**Figure 2:**
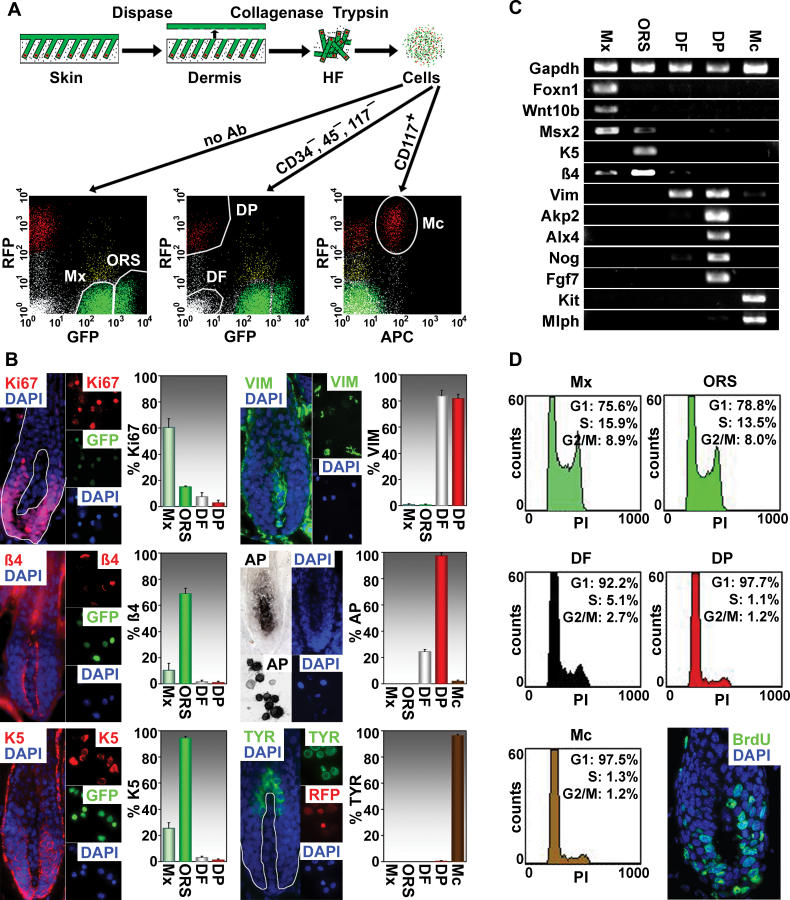
Isolation and Purification of Mx, ORS, DP, DF, and Mc Populations (A) Schematic of isolation procedure. After removing subcutaneous fat by dissection, and epidermis/upper follicle segment by enzymatic digestion, single-cell suspensions were prepared from pure dermis and subjected to three FACS schemes to purify five populations of cells: Mx, GFP^low^RFP^−^; ORS, GFP^high^RFP^−^; DP, RFP^high^GFP^−^CD34^−^CD45^−^CD117^−^; DF, RFP^−^GFP^−^CD34^−^CD45^−^CD117^−^; Mc, RFP^high^GFP^−^CD117^+^. (B) Immunofluorescence analyses of FACS isolated cell populations. Frozen skin sections (hair bulb) and relevant cytospin populations were stained with Abs as color-coded and indicated. At the right of each set is quantification of percentage of cells that expressed the marker. Note: ~10% of DP and DF cells lysed on cytospin. and hence did not stain with any markers. β4, β4 integrin; Tyr, tyrosinase; Vim, vimentin; white line, basement membrane. (C) RT-PCR: cDNA fragments were resolved by agarose gel electrophoresis, and the gene detected is denoted at left. All fragments were of the expected size. Expression of Msx2, vimentin, and β4 in multiple populations was later confirmed. (D) Cell cycle differences in cell populations. Profiles of the five purified populations were performed by FACS. Anti-BrdU immunofluorescence is from a P4 backskin follicle from a mouse injected intraperitoneally with 50 μg/g 5-bromo-2′-deoxyuridine (BrdU) (Sigma-Aldrich) and analyzed 4 h later. Note greatest incorporation in Mx and ORS.

During embryogenesis, hair follicle formation is dependent upon a series of reciprocal interactions between the single-layered epithelium and a dermal cell condensate. This specialized cluster of mesenchymal cells becomes enveloped by the epithelial (matrix [Mx]) cells at the base of the developing follicle, and postnatally, they persist as the dermal papilla (DP) (Figure 1A; [1,2] ).

The architecture and biology of the mature follicle is complex (Figure 1A). At the base and in close association with the DP, Mx cells are transiently proliferative and maintain a relatively undifferentiated status. As Mx cells progress upward, they differentiate into the hair shaft (cortex and medulla) and the channel or inner root sheath (IRS) that surrounds the hair. The IRS is then encased by an outer root sheath (ORS) contiguous with the epidermis. The entire structure is enclosed by a basement membrane composed of extracellular matrix (ECM) proteins that separate the skin epithelium from the dermis and DP. A small number of follicle melanocytes (Mc) reside just above this membrane in the epithelial compartment of the hair bulb.

When Mx cells exhaust their proliferative capacity, the hair stops growing, and the lower epithelial part of the follicle enters a destructive phase (catagen). As the epithelium shrinks, the basement membrane and DP move upward. Following a resting period (telogen), epithelial stem cells (SCs) at the base of the remaining hair follicle (the bulge) receive signals from the now adjacent DP, and reenter a growth phase (anagen) to regenerate the follicle and produce a new hair.

Genetic engineering has recently enabled the isolation of epithelial SCs within the bulge [3,4]. When exposed to skin dermis, the descendants of a single epithelial SC can give rise to epidermis, follicles, and sebaceous glands, when engrafted onto the backs of *Nude* mice lacking hair [5]. It has long been recognized that the critical mesenchymal cells in this process are the DP [1]. In contrast to dermal skin fibroblasts (3T3 cells), which only permit epidermal repair in this assay, microdissected rat whisker DP cells induce hair growth [6,7]. In vitro, the DP cells lose this ability. Co-culturing DP cells, either with epidermal keratinocytes [8], or with embryonic fibroblasts expressing a *Wnt3a* but not a Sonic hedgehog *(Shh)* transgene [9], prolongs their potential.

Knowledge of the genes expressed by the DP and its neighbors would be of obvious value in sifting through the complex mechanisms by which DP cells maintain their remarkable inductive function while in the niche, and lose them outside of it. To date, most known DP markers have been found fortuitously. Because the DP resides in the center of a diverse cellular niche, comprised of surrounding Mx and ORS cells, Mc, and dermal sheath cells, its relative inaccessibility, coupled with its loss of potential in vitro, have posed major technical hurdles in cleanly isolating populations of these cells. Thus, although microarray and cDNA library analyses have been conducted on microdissected and/or cultured whisker DP [10–12], the array data have yielded only a handful of the known DP markers, making it difficult to evaluate the potential significance of unexpectedly expressed genes from these arrays.

Recently, it was proposed that the DP might be the origin of multipotent skin-derived precursor cells (SKPs), which are cell aggregates derived from skin cultures [13]. Interestingly, SKPs bear some resemblance to neurospheres derived from cultured neural SCs, and in vivo, a few SKP markers localize to DP. This has led to the speculation that the DP might be the residence for neural progenitor cells [13]. However, these analogies are complicated by the close proximity of Mc (neural crest derived) and DP in the follicle. Additionally, in contrast to other body sites, the head dermis develops embryologically from neural crest [14], and the parallels are drawn largely from studying rodent whiskers [13]. Thus, although the existence of a population of multipotent neuroprogenitor cells in adult follicles would place the DP squarely at the center of major clinical relevance, it remains unclear as to just how similar DP cells actually are to neuronal cells.

To probe more deeply into the special features of the DP and the nature of their cross-talk with neighboring cells, we have developed a novel strategy employing double-transgenic mice, in combination with selective cell-surface labeling to facilitate the purification of backskin DP cells and the cells surrounding their niche. By employing fluorescence activated cell sorting (FACS), we purified sufficient quantities of DP and four additional cell populations to obtain their transcriptional profiles. This has allowed us to identify the defining features that distinguish the DP cell from its neighbors, including Mc, and also the epithelial progenitors that receive cues from the DP to give rise to the differentiated cells of the hair shaft and its channel. With these molecular signatures, we have gained new insights into the DP and its microenvironment. These analyses now pave the way for future dissection of the key inductive signals produced and received by the DP that are lost upon culture in vitro. In addition, the novel multicolor labeling strategy and rigorous cross-comparisons between multiple, closely interacting cell types should have broad applicability in deciphering which genes within microarrays are likely to play key functional roles within a complex cellular niche or tissue.

## Results

### Isolation and Purification of DP and Four Neighboring Cell Types/Lineages

The DP cells are underrepresented dermal residents that exist as the cellular nut cloaked by a microenvironment composed of other cell types. We therefore devised a novel strategy that would enable us to use FACS to purify the DP from its complex cellular surroundings. We engineered transgenic mice expressing red fluorescent protein (RFP) under the control of a human Lef1 promoter fragment [15], and mated them to mice expressing histone H2BGFP under the control of a keratin 14 (K14) promoter [4] (Figure 1B). The K14 promoter is active only in the epithelial cells of the skin [16]. Because its activity includes epithelial SCs, H2BGFP was detected in all of the follicle epithelial nuclei. However, the promoter is most strongly active in the transiently amplifying cells of the basal epidermal and ORS layer, and correspondingly, this is where the H2BGFP was most abundant (Figures 1C and S1). By contrast, H2BGFP levels were approximately 3× reduced in Mx cells, and in differentiated Mx progeny (IRS, hair shaft, companion layer) (Figure S2). These data were consistent with the marked downregulation of K14 promoter activity upon Mx cell specification [17].

In marked contrast to the H2BGFP expression pattern, cytoplasmic RFP levels were strongest in the DP (Figure 1B and 1D). The only other location of strong RFP in the skin was in the Mc, typified by their co-expression of tyrosinase (Figure 1D) and CD117 (*Kit;* unpublished data). Interestingly, RFP was not found in the Mx or precortex cells where the endogenous murine *Lef1* gene is normally expressed [18]. Weak RFP was sporadically found in the premedulla, which was also positive for H2BGFP (double-positive FACS population in Figure 2A).

To isolate follicles, we first treated P4 backskins with dispase to selectively remove and discard the epidermis and uppermost parts of hair follicles, and then digested the dermal ECM with collagenase (Figure 1B). After brief trypsinization, larger debris (including hair shafts) was removed by passing the cell suspension through a cell strainer, eliminating the green fluorescent protein (GFP) positive, terminally differentiated hair cells that were still largely attached to the hair shaft. The single-cell suspension was then subjected to three different FACS isolation schemes. Channels specific for GFP and RFP were employed in various combinations with antibodies (Abs) against different cell surface markers to isolate Mx, ORS, Mc, DP, and a dermal fraction (DF) enriched in fibroblasts (Figure 2A). Prior to FACS, we stained with the cell surface marker Abs to verify that these markers were not lost by the trypsinization procedure (unpublished data).

The ORS and Mx were sorted based on their 3-fold different levels of GFP expression and absence of RFP (ORS: GFP^high^RFP^−^, Mx: GFP^low^RFP^−^). For DP and DF isolation, whole-cell preparations were first subjected to immunolabeling and magnetic depletion of RFP-positive Mc (CD117) and of dermal endothelial cells (CD34) and immune cells (CD45). The fractions were then sorted as the RFP-positive (DP) and -negative (DF) fractions, and further distinguished by their absence of GFP. Thus, DP cells were RFP^high^GFP^−^CD34^−^CD45^−^CD117^−^, while DFs were considered as those cells that were RFP^−^GFP^−^CD34^−^CD45^−^CD117^−^. Finally, Mc cells were selected as the RFP- and CD117-positive population in a separate immunolabeling (Mc: RFP^high^GFP^−^CD117^+^).

We judged the purity of each population by immunofluorescence microscopy and RT-PCR analyses (Figure 2B and 2C). As predicted, the putative Mx fraction showed strong labeling with Abs against proliferating nuclear antigen Ki67 and weak labeling with Abs against keratin 5 (K5) and K14 (Figure 2B). Included mRNAs in this population were *Wnt10b, Msx2,* and *Foxn1,* known to be expressed in the Mx (Figure 2C). In contrast, less than 7% of the cells in this sorted population labeled with markers characteristic of the differentiating Mx progeny (Figure S2). This was true for the hair cortex (hair keratins, AE13), the IRS/medulla (trichohyalin, AE15), and the companion layer/medulla (a K6 Ab diagnostic for these cells) (Figure S2). These data corroborated our purification strategy for the Mx. The putative ORS fraction appeared to be similarly pure and distinct from the Mx pool. Thus, the ORS population was strongly positive for K5 and β4 integrin, and displayed reduced Ki67 and *Msx2* and no detectable *Wnt10b* (Figure 2B and 2C).

We examined the purity of the CD117, RFP-positive, and GFP-negative Mc fractions by testing for tyrosinase, *Kit*, and melanophilin—three key Mc markers. These markers were present in the Mc fraction, but they were not detected in the other populations (Figure 2B and 2C).

We were particularly interested in the DP and in defining its unique features that distinguish these cells from dermal fibroblasts. As expected, our DP and DF fractions were both enriched for vimentin, but alkaline phosphatase (AP) activity was strong only in the DP (Figure 2B). Nearly every cell in the DP fraction exhibited some AP activity, and ~90% displayed very strong activity (Figure 2B and Figure S3A and S3B). Some AP activity was detected in the DF, which could be due to the known presence of low AP activity in some of the non-DP dermal sheath cells, at the base of the hair follicles [19]. As judged by semi-quantitative RT-PCR, the level of AP mRNAs (*Akp2*) was markedly higher in the DP than the DF fractions (Figure 2C). Moreover, only the DP fraction scored strongly positive for mRNAs encoding the additional known DP markers *Alx4*, Noggin, and *Fgf7* (Figure 2C). This preliminary molecular analysis suggests a purity of this DP fraction not achieved by previous methods [9–12].

Finally, we verified functionality of the DP fraction by culturing them in vitro for 1–2 wk, and then grafting the cultured DP cells with keratinocytes onto the backs of *Nude* mice. In contrast to grafting either keratinocytes alone or keratinocytes in conjunction with cultured dermal fibroblasts [7], the DP fraction produced haired skin (Figure S3C). Such characteristics have only been ascribed to DP cells or so-called dermal cup cells at the base of the hair bulb [9,19]. These functional data lend further evidence of the DP character of our population.

The cell-cycle profiles of the five populations varied in accordance with the levels of anti-Ki67 labeling and BrdU incorporation in vivo (Figure 2B and 2D). Quiescent DP and Mc populations displayed less than 1% cells in S-phase. The transiently dividing populations of Mx and ORS showed ~15% of S-phase cells. When coupled with protein and mRNA expression patterns, the specificity of cell cycle profiles further validated the purification schemes and confirmed the identity of each fraction.

### Molecular Signatures of the DP and its Four Neighboring Populations

**Figure 3:**
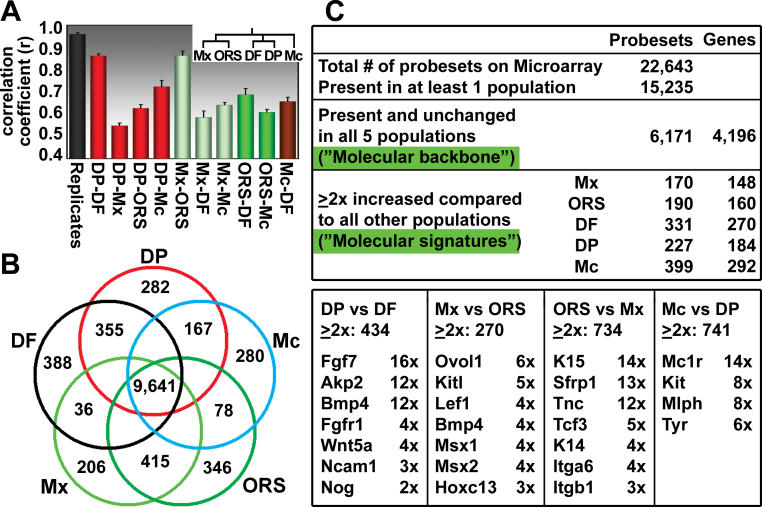
Gene Expression Patterns of the Five Hair Follicle/Skin Populations (A) Overall correlation of gene expression profiles between different cell types. A high correlation coefficient was observed between the DP-DF and Mx-ORS populations, consistent with their mesenchymal versus epithelial characters. Intriguingly, the least similar populations were the Mx and DP, which are strongly engaged in reciprocal exchange of signaling necessary for the maintenance of both compartments. Inset: Dendrogram outlining the correlation of gene expression between the populations. (B,C) The Venn diagram demonstrates the degree of similarity based on absolute present calls. Probe-sets were considered present only if they were called present in both replicates. Note that for each cell type, ~200–400 probe-sets showed selective hybridization under these criteria, while ~2/3 of all present probe-sets were represented in at least four or all five of the fractions and ~4,000 genes (~6,000 probe-sets) were expressed and unchanged in all five populations (“Molecular backbone”). When all five fractions were cross-compared, ~150–300 genes (“Molecular signatures”) scored as being upregulated by ≥ 2× selectively in only cell type. The lists show array predictions for differential expression of mRNAs encoding some established, defining markers for each cell type, when compared against an adjacent or related cell population. The total number of mRNAs that show at least a 2× difference between the two cell populations is provided at the top, and the fold difference in specific mRNA levels is given next to each marker. DF, dermal fraction; DP, dermal papilla; Mc, melanocytes; Mx, matrix; ORS, outer root sheath.

By purifying all of the cell populations within the niche of the hair bulb, we were able to obtain the transcriptional information necessary to dissect the commonalities and differences of these cell types, both at a global and at a gene-by-gene basis. For each population, purifications and microarray hybridizations (Affymetrix Moe430A) were performed in duplicate. A high level of correlation (96 ±0.7%) between replicate hybridizations (Figure 3A and Table S1) and other quality-control statistics validated our performance of microarray data generation (Table S2). Raw data and normalized microarray expression data can be accessed at the Gene Expression Omnibus (http://www.ncbi.nlm.nih.gov/geo/). A cursory examination of the overall correlation of genes present in each fraction revealed the relationship of each cell population relative to the other cell types of the niche (Figure 3A). The corresponding dendrogram allowed further visual inspection of the relations between the five different cell populations of the DP niche (Figure 3A, inset). While the correlation between replicates set the standard of a near perfect match (*r* > 0.96), there was a remarkably high correlation between the DP and DF, and the Mx and ORS, respectively, highlighting the common mesenchymal origin of DP and DF and the close lineage relationship of Mx and ORS. Intriguingly, the lowest correlation occurred between DP and Mx, revealing striking differences between the two populations whose signaling exchange orchestrates the dynamics of the hair growth.

We next turned to high-stringency comparative analyses to uncover their common and distinguishing features. Initial inspection of the distribution of genes called “present” irrespective of their expression levels revealed that more than two-thirds of the more than 22,000 probe-sets scored as present in at least one population (Figure 3B), with the bulk of genes being present in at least four or all five fractions. Conversely, a few hundred genes were present exclusively in one fraction, and the number of genes present in the overlap between any two fractions again highlighted the close lineage relationship of Mx and ORS, and DP and DF, respectively (Figure 3B). To ensure that we did not overlook genes that are called present in more than two fractions and yet show dramatically different expression levels (e.g., 4× present: 3× < 200, 1× > 2,000) we next performed comparative analyses, providing a more robust measure of the commonalities and differences between the five cell populations. Of the more than 9,000 probe-sets called present in all fractions, ~6,000 (4,000 genes) scored also as unchanged, comparing all five populations against each other, providing a list of putative housekeeping or “molecular backbone” genes irrespective of the lineage or cell type (Figure 3C and Table S3). By contrast, only 150–300 genes scored as upregulated by at least 2-fold in one fraction relative to the other four. In many cases, these genes were also selectively called present in only one of the fractions indicative of a specialized function. These subsets provided “molecular signatures” for each population (Figure 3C).

Each signature faithfully contained many previously assigned markers for each cell type and differentiation status [2,20]. In addition, the arrays permitted comparisons of relative expression levels of these genes in different cell compartments (Figure 3C). The mRNA level for the Mx growth factor *Fgf7* was more than 16× higher in DP than DF. The mRNAs encoding known transcriptional regulators of Mx cell growth and differentiation were 4–6× higher in Mx than ORS. Conversely, mRNAs encoding ORS keratins were 3–15× higher in ORS versus Mx. mRNAs required for melanin pigment granule production were 6–14× higher in Mc than DP. Comprehensive lists of all signature genes are provided in Tables S4–S7.

**Figure 4:**
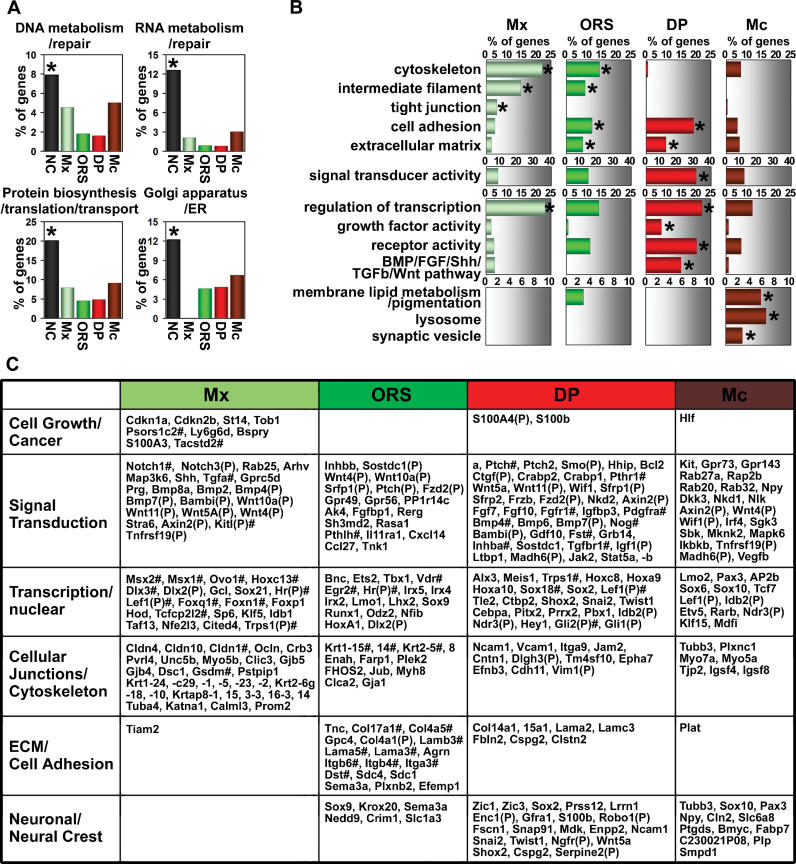
GO Analyses and Functional Grouping of the Molecular Backbone and Signatures (A) GO analyses of ~4,000 genes present and unchanged in all five fractions irrespective of lineage or cell type. Shown is the percentage of genes in a given GO category, compared to all genes of the signature of a given cell type. Note that the genes were enriched mostly in categories involved in basic cell functions representing the molecular backbone. Asterisks denote a significant increase over a whole genome prediction. NC, not changed. (B) GO analyses of the molecular signatures. The signature was defined as the genes whose expression was upregulated by ≥ 2× in only one of the five hair/backskin populations. Each signature was categorized into groups of genes depending upon their putative cellular functions. Shown is the percentage of genes in a given GO category, compared to all genes of the signature of a given cell type. Asterisks denote a significant increase over a whole genome prediction. (C) The molecular signatures. The gene abbreviations and/or accession numbers are according to the NCBI listings. # denotes genes implicated in skin/hair disorders. (P) denotes genes with appreciable signal but higher levels in one of the other four populations. For multiple genes in a signature, the abbreviation is listed once, followed by –x, where x is the specific gene number.

The presence of the expected cell type–preferred patterns of gene expression gave us the confidence to progress to novel features of the signatures. Although we used the DF fraction for comparative purposes, we concentrated on the four populations at the base of the follicle. We grouped their signature genes and the list of common, unchanged genes (molecular backbone) into putative functional categories based upon established Gene Ontology (GO) classifications (http://www.geneontology.org/) and calculated significantly enriched categories (Figure 4A and 4B and Table S8). The common, unchanged group was largely genes encoding proteins involved in basic cellular functions, such as DNA, RNA, and protein metabolism (Figure 4A). A complete list of all GO classification of the molecular backbone is provided in Table S9. Semi-quantitative RT-PCR analyses verified that these mRNAs were expressed at similar levels across the five cell populations (Figure S4).

In contrast, the differential and/or overlapping enrichment of genes in the specialized categories of the signatures provided the first insights, at a genomic level, of the functional properties of the different niche cell types (Figure 4B). Genes within the most relevant categories are listed in Figure 4C. The signatures contained many novel genes associated with signal transduction pathways of hair follicle morphogenesis, cell type−specific transcriptional regulators, cytoskeletal components, and ECM and adhesion molecules (Figure 4B and 4C). A detailed list of significant GO categories for each signature is provided in Table S8, and comprehensive signature gene lists sorted by GO classifications can be found in Tables S10–S13.

**Figure 5:**
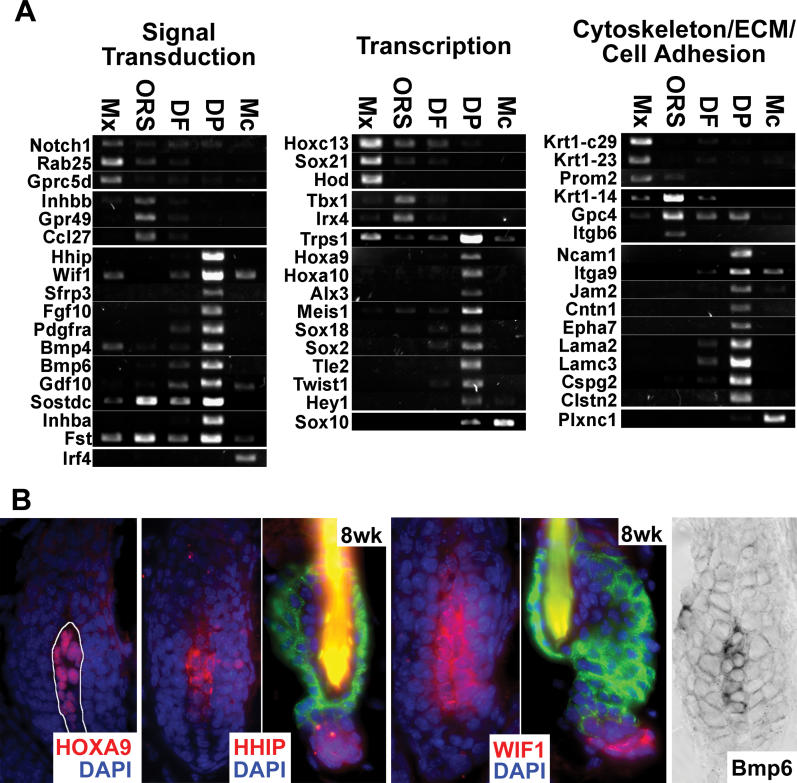
Implementation of Array Analyses to Examine Characteristics and Dynamics of the Follicle DP Niche (A) Semi-quantitative RT-PCR on mRNAs isolated from each population. Shown are representative data from molecular signature genes (see Figure 4) whose expression patterns in the DP niche environment had been previously uncharacterized. In this case, categories were consolidated into three groups: Signal transduction, transcription/nuclear, and cytoskeleton/ECM/cell adhesion. For each primer set, at least three different cycles were employed, and the resulting cDNA fragments were resolved by agarose gel electrophoresis along with DNA size markers to confirm that bands were of the expected sizes. For each gene, the data presented were from the cycle that provided the most meaningful comparisons. Note: bands seen in > 1 fraction accurately reflect mRNA expression at the differences in levels shown. (B) Immunohistochemistry and in situ hybridizations. Skin sections were taken from 2-mo-old K14-GFPactin mice [49] whose follicles were at the transition from the resting to growing (telogen to anagen) stage of the hair cycle (8 wk) or from P4 WT mice (full anagen follicles) (all others). Sections were subjected to either immunofluorescence using color-coded Abs as indicated or in situ hybridization using the indicated biotinylated cRNA probes (sense controls were negative).

To rigorously test the degree to which the microarray data faithfully recapitulated the unique expression patterns of each cell type, we performed a series of semi-quantitative RT-PCR (Figure 5A) and real-time PCR analyses (Figure S5) across all five cell populations. For these analyses, we selected a number of mRNAs encoding signaling molecules, transcription factors, and ECM/adhesion molecules that scored as preferentially upregulated in one of the populations relative to the others (Figures 5A and Figure S5). We then contrasted the fold changes of the real-time PCRs with the actual average signal values of the microarray analyses. As shown in Figure S5, the expression patterns were remarkably similar, and often indistinguishable. These data provided a graphical illustration of the degree to which the comparative analyses based upon our microarray analyses faithfully recapitulated the differential expression patterns of the signature genes within the DP niche. Overall, these expression patterns should be helpful in future studies aimed at understanding how these genes play functional roles in hair biology. Below, we highlight some key features of the signatures.

### The ORS and Mx Signatures

The ORS signature included genes encoding a complex array of largely unstudied putative skin transcription factors. This list contained known *(Bnc, Ets2, Tcf3, Egr2/Krox-20,* hairless *[Hr],* and vitamin D receptor *[Vdr]),* as well as previously unrecognized ORS transcription factors (Figures 4B, 5A, and Figure S5). The signature was further distinguished by focal adhesion and ECM genes, reflecting an ability of ORS cells to not only to adhere to, but also synthesize and remodel their adjacent basement membrane. Since ECM is composed of signaling molecules, the upregulation of these genes further suggested a possible feedback loop to reinforce cell-substratum contacts in the ORS.

In contrast to ORS, Mx cells are typified by their ability to respond to cues from their microenvironment and differentiate upward to form the six concentric rings of the hair follicle. The Mx signature revealed their status at the nexus of proliferation and differentiation (Figures 4B and S5). In addition to established Mx transcription factors *(Msx2, Msx1, Ovol1, Hoxc13, Dlx3, Foxn1, Hr, Lef1,* and *Ap2),* the signature included several forkhead cousins of *Foxn1* (*Nude* mouse), one of which (*Foxq1*) has been linked to the *Satin* mutant mouse, defective in hair shaft differentiation [21]. Also on this list were *Gcl* (germ cell-less) and *Tcfcp212* (grainyhead-like1), thought to function in early SC differentiation and/or lineage boundaries. The Mx signature also revealed many genes encoding members of the *Fgf, Wnt, Tgfβ, Tgfα, Shh,* and *Bmp* signal transduction pathways (Figure 4C). This was in good agreement with the established ability of Mx to orchestrate signal transduction pathways and specify the hair shaft and its channel. Additionally, the signature included genes encoding keratins and other structural proteins. In part, this could reflect early steps in lineage differentiation. However, for at least three structural genes, it is noteworthy that (a) keratin c29 is highly homologous to K17, whose absence causes premature Mx apoptosis and alopecia in mice [22]; (b) skin lacking *Cldn1* (claudin 1) displays abnormally short hairs [23]; and (c) *Gsdm* (gasdermin) mutations have recently been linked to alopecia in mice [24].

### The DP Signature: Insights into Mesenchymal–Epithelial Cross-Talk

A goal of this study was to identify novel features of the DP that might give us insights into understanding how these cells exert their power over epithelial SCs and their ORS and Mx progeny. By comparing against DF, we screened out general fibroblast features, e.g., expression of type I and type III procollagen chains, vimentin, and *TGFβ1*-induced genes. By contrasting the DP with ORS and Mx signatures, we could identify genes exclusively expressed in either compartment and begin to make predictions regarding the epithelial–mesenchymal cross-talk that transpires in the hair bulb.

The purity of our DP cells yielded an unprecedented sensitivity of detection. Of approximately 30 genes reported to be expressed in DP in vivo [25], 24 were either in our DP signature or expressed in DP but more abundant in one or more of the other populations (Figure 4C). By contrast, only three of these genes had appeared on the prior array list from microdissected DP [11], and only five were on the list of 309 expressed genes from cultured DP [12]. Most of the ~180 genes in our DP signature encoded novel factors involved in transcription, cell communication, and signaling (Figure 4C). Less than 5% of our DP signature genes appeared on the previously published arrays of microdissected whisker DP in vivo [11] or in vitro [12].

Given the near complete lack of overlap between our DP signature and prior published reports, it was important to verify the novel aspects of each signature, as we had already done for the well-established features. Semi-quantitative PCR confirmed that the majority of genes were expressed predominantly by only a single cell population, i.e., the hallmark of our signature lists (Figure 5A). The few exceptions were readily explained upon inspection of the gene expression profiles across the five populations. For example, *Fst* (follistatin) and *Sostdc1* (ectodin/wise) scored as ~3× higher in DP than in ORS, but 3–30× higher in ORS than in the other three fractions. Analogously, *Wnt5a* and the Gata 3-like factor *Trps1* (tricho-rhino-phalangeal syndrome1) scored as ~2–6× higher in DP than in Mx, respectively, but ~1.5–10× higher in Mx than in other fractions. Real-time PCR further documented the accuracy of the DP signature (Figure S5).

Finally, we showed that expression of our DP signature genes can be detected in highly enriched pelage follicle preparations (see below). For a number of novel DP genes, we also used in situ hybridization and immunofluorescence to verify mRNA expression patterns and extend our findings to the protein level (Figure 5B). That our DP signature bears strong resemblance to the list of known DP genes and bears little resemblance to previously published profiles of DP cells emphasizes the importance of conducting array analyses on purified populations of skin DP cells. The PCR, in situ hybridizations, and immunofluorescence data offer compelling evidence to attest to the faithfulness and reliability of our signatures, and provide the first clear view of the DP and its niche microenvironment.

### Functional Links Between Array Data, Human/Mouse Genetic Disorders of Hair and Skin, and Epithelial–Mesenchymal Interactions in the Hair Follicle

**Figure 6:**
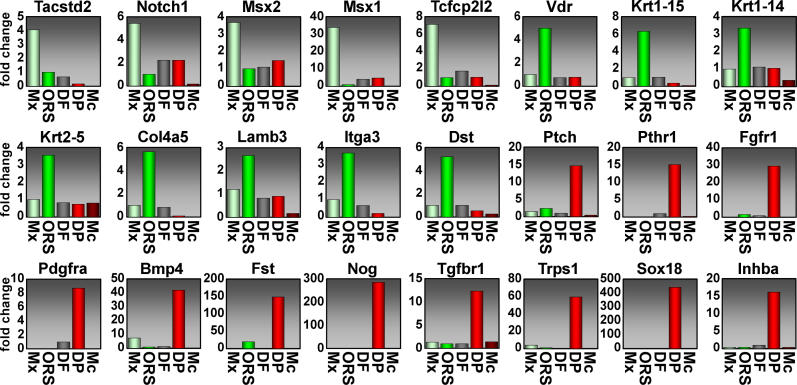
Detailed Expression Analysis of Hair/Skin Disease Genes Found in the Molecular Signatures of the Epithelial and Mesenchymal Populations of the Hair Bulb Real-time PCR confirmation of 24 different signature genes of the Mx, ORS, or DP, which have previously been implicated in genetic disorders of the hair. Many of these genes have not been well-studied at the level of expression and function. In each case, the highest level of mRNA expressed corresponded to the cell population in which the signature gene appeared. Moreover, in cases where more than one cell population showed appreciable mRNA levels, this was also reflected in our microarray comparisons.

Our array comparisons provided us with the confidence to probe more deeply into the physiological relevance of the signature lists. One of the most interesting and striking features of our array comparisons was the large number of signature genes that are associated with different genetic disorders of the hair. Denoted by a “#” in the signature lists of Figure 4C, these genes included (a) the Mx signature genes *Psors1c2, Tacstd2, Notch1, Msx2, Msx1, Hoxc13, Dlx3, Foxq1, Tcfcp212, Trps1, Hr, Cldn1,* and *Gsdm;* (b) the ORS signature genes *Pthlh, Vdr, Egr2, Hr, Krt1–15, Krt1–14, Krt2–5, Col17a1, Col4a5, Lamb3, Lama5, Lama3, Itgb6, Itgb4, Itga3,* and *Dst;* and (c) the DP signature genes *Ptch, Pthr1, Fgfr1, Pdgfra, Bmp4, Fst, Nog, Tgfbr1, Trps1, Sox18,* and *Inhba*. In addition, the hair disorder–associated genes included several genes, e.g., *Kitl, Lef1, Hr,* and *Gli2*, which were featured prominently in the arrays, but which were expressed at relatively high levels in more than one of the five cell populations, thus excluding them from the signature lists. Real-time PCR was used to confirm the expression patterns of these functionally important signature genes (Figure 6).

Even though these genes have been previously genetically linked to hair/skin disorders, only a few have been well-studied at the level of expression and function. We were particularly intrigued by DP signature genes such as *Trps1, Sox18, Fst,* and *activinβ-A (Inhba),* whose roles in hair follicle morphogenesis have remained poorly understood [26–29]. Of additional note was the DP signature gene *Fgf10,* recently shown to be required for embryonic whisker development [30]. *Fgf10* and *Fgf7* bind to the same receptor (encoded by *Fgfr2* and in the Mx signature), and *Fgf10′s* presence in the DP signature explains why *Fgf7* knockout mice display a milder hair phenotype than the conditional *Fgfr2* knockout [31,32].

Further insights into the DP-Mx cross-talk came from evaluating the distribution of Shh pathway members. Whereas *Shh* is expressed by Mx, Shh receptor and downstream effector genes were part of DP's signature (Figure 4C). Additionally, mRNA encoding *Hhip* (hedgehog-interacting protein) was more than 80× higher in DP than Mx (Figures 5A and S5). By in situ hybridization and anti-Hhip immunofluorescence, we detected *Hhip* at the early stages of follicle downgrowth (Figure 5B). This was intriguing since in lung development, *Shh* signaling through Patched can accentuate *Hhip* expression, making the extending lung bud tip refractory to *Shh* signaling and permissive for *Fgf10* expression [33]. Moreover, *Fgf10* is known to be negatively regulated by Shh, and conversely, both mesenchymal *Fgf10*, and also the BMP inhibitor Noggin, can enhance epithelial *Shh* expression [34,35]. When taken together, our findings suggest a regulatory circuitry for sustaining expression of *Fgf10/7* in Hhip-positive DP and permitting *Shh* in Mx. Since excess *Shh* would be expected to override the effects of Hhip and downregulate *Fgf10* and *Fgf7,* this may also explain why Shh treatment per se did not maintain the inductive ability of cultured DP [9].

Given the reported effects of Wnts on the maintenance of DP potential [9] and the presence of *Wnt5a* in embryonic dermal condensates [36], it was interesting that *Wnt5a,* previously reported in postnatal hair follicles [36], was in the DP signature (Figure 4C). Equally intriguing was the presence of DP signature genes encoding both secreted Wnt inhibitors *(Wif1, Sfrp2,* and *Frzb),* as well as possible Wnt effectors. Semi-quantitative RT-PCR and real-time PCR (Figures 5A and S5), as well as anti-Wif1 immunofluorescence supported these observations (Figure 5B). Like *Hhip*, *Wif1* expression was maintained in adult DP and present at different stages of the hair cycle.

Of all the novelties in the DP signature, we were particularly struck by the number of *Bmp* pathway members whose mRNA expression levels were upregulated by at least 2× in DP. *Bmp4* has already been implicated in the cross-talk that specifies hair differentiation [37]. However, *Bmp6* was particularly notable in that its mRNA levels were more than 10× higher in DP than the four other populations, a feature confirmed by in situ hybridization (Figure 5A and 5B, and Figure S5). All the cells within the hair bulb, including the DP, expressed the requisite BMP receptor (*Bmpr1a*). This said, the DP signature included a surprising number of genes encoding BMP inhibitors, including *Noggin, Gdf10, Sostdc1/Ectodin/Wise* [11], *Prdc* (protein related to Dan/Cerberus), and *Bambi*. Of these, only *Noggin* has previously been documented as a functionally important BMP inhibitor in the DP [34,38]. The preponderance of BMPs/BMP inhibitors in the DP signature suggested a greater importance of the BMP pathway in promoting DP character than had been previously appreciated.

### The DP Signature: Relation between Neural SCs and DP Cells

Recently, it was reported that skin cultures contain neurosphere-like structures that can be induced to form neurons and glial cells [13,39]. Although prior array data on dissected whisker DP, and their cultures, showed no resemblance of DP to neurally derived cells [11,12], several markers expressed by the skin-derived neurospheres were traced by in situ hybridization to whisker follicles [13]. The relative lack of resemblance between these prior whisker “DP” screens and our signature containing bona fide DP markers offered a possible explanation for these discrepancies. However, since some of head mesenchyme is known to be derived from neural crest [40], a documented resemblance between whisker DP and neural progenitor cells would still not be definitive. Our array data allowed us to address more important and as yet unexplored questions: (1) Do SKPs and/or neural progenitors share similarities with DP from skin whose mesenchyme is not derived from neural crest? and (2) How does DP character compare to that of neural progenitors, nearby Mc (of known neural crest origin), and dermal fibroblasts (derived from dermamyotome)?

**Figure 7:**
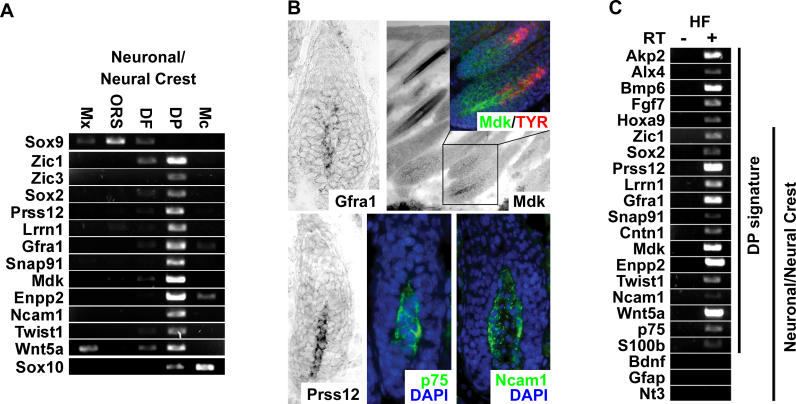
Neuronal and Neural Crest Genes Expressed by the DP and Hair Follicles (A) Semi-quantitative RT-PCRs were conducted as in the legend to Figure 5, except in this case, we used oligonucleotides against neuronal and/or neural crest expressed genes. In all cases, these genes either appeared on the DP molecular signature or scored as expressed by the DP as well as one or more of the other four skin populations. (see Figure 4) Shown are representative RT-PCR data, which show an excellent correlation with the DP-preferred expression pattern of the majority of these neural genes. (B) Immunohistochemistry and in situ hybridizations of neuronal/neural crest genes in skin. Sections of P4 backskins were subjected to either immunofluorescence using color-coded Abs as indicated or in situ hybridization using the indicated biotinylated cRNA probes (sense controls were negative). Merged images of serial sections were used to compare Ab (red) and in situ (pseudogreen) patterns. *Gfra1*, glial derived neurotrophic factor receptor1; *Mdk*, midkine; *Prss12*, serine protease neurotrypsin; Tyr, tyrosinase. (C) Detection of neuronal/neural crest genes in highly enriched hair follicle preparations. Highly enriched follicle preparations were isolated by serial low-speed centrifugation following dispase and collagenase digestion of P4 backskins. After isolation and preparation of their mRNAs, semi-quantitative RT-PCR was conducted using oligonucleotides to those neuronal and neural crest markers that were found in the DP signature. As controls, oligonucleotides were used against *Akp2*, *Alx4*, *Bmp6*, and *Fgf7*, which are all markers that we mapped to DP by in situ hybridizations and/or immunofluorescence (see Figure 5). Note that the neuronal/neural crest genes appearing on the DP signature showed comparable signals to the documented DP genes.

We first addressed the relation between DP and SKPs cultured from skin dermis [13]. Only five genes, *Snai2* (slug), *Twist1, Cspg2* (versican), *Nexin1,* and *Ncam1,* have been reported to be expressed in both SKPs and backskin follicles [9,13,41,42]. Four of these genes appeared on our DP signature (Figure 4C). Of the remaining known SKP-expressed genes (*Shox2, Pax3, Snail1, Sox9, Nestin, Wnt-1, Sca-1/Ly6A-E, Twist2,* and *Fn1)* [13], only *Shox2* was in the DP signature, and only *Fn1* scored as present in DP. Conversely, *Sox2* and *Ngfr (p75)* were readily detected in pelage DP (Figure 7) and yet they were reported as absent in SKPs [13].

Although differences between SKP cultures and in vivo DP expression patterns had escaped prior notice, such differences could nevertheless exist because SKPs are derived from cultures rather than a purified in vivo cell population. We therefore turned to addressing the broader relation between DP and neural SCs. In this regard, it was notable that *Zic1, Zic3,* and *Sox2* were all part of the DP signature and absent in Mc. These mRNAs encode key transcription factors that specify neuronal fate at the expense of ectoderm [43,44]. The signature also included about ten other neural genes (Figure 4C).

We confirmed the preferred expression of these genes in DP by using semi-quantitative RT-PCR. As shown in Figure 7A, most genes were preferentially upregulated in the DP fraction relative to all of the other fractions, including Mc. An exception was *Sox10,* whose expression by array analyses and by RT-PCR scored as preferentially expressed in Mc. Also confirming the array data were our RT-PCR analyses of *Sox9,* which scored as preferentially expressed in the ORS, and *Wnt5a,* which scored as present in Mx and DF populations as well as in the DP (Figure 7A). We also confirmed DP localization of *Prss12 (serine protease neurotrypsin), Gfra1 (glial derived neurotrophic factor receptor1),* and *Mdk (midkine)* by in situ hybridization (Figure 7B). Co-labeling with anti-tyrosinase (Mc-specific) verified that the hybridization was in the DP and not Mc compartment. In addition, we verified the expression of these and additional neuronal/neural crest–related DP signature genes in highly purified follicle preparations (Figure 7C). Given the in vivo expression of neuronal/neural crest–related mRNAs in the DP, we could not attribute the unusual expression patterns to the presence of minor neural contaminant(s), e.g., Schwann cells, which are likely to ensheath the sensory nerve endings within the skin [45]. Rather, the in situ and immunofluorescence patterns of the neuronal component of the DP signature (Figure 7) showed a good correlation with the physical location of the DP, as did the in vivo expression pattern of the DP signaling and follicle disease genes.

Despite the alluring parallels between backskin DP and cells of neural origin, the DP signature did not strongly resemble neural crest, neural SCs, or any of the neural lineages described to date, including Mc. Additionally and equally surprising was the degree to which the DP and dermal fibroblast signatures were distinct, as the DF signature did not display these neuronal-like parallels, nor did they exhibit the bank of hair disease genes or signaling genes seen in the DP signature. Taken together, our data point to a signature unique to the DP and not shared by any of the cell populations constituting the distinctive DP niche microenvironment.

## Discussion

The potent inductive ability of DP to promote follicle formation has been recognized for decades [1]. However, their minority status, coupled with their rapid loss of potential in vitro, has left their molecular nature elusive. By devising a strategy to obtain pure DP and their neighboring cells, we were able to overcome this hurdle and determine a molecular signature for DP. By comparing expressed DP genes to those of DFs, Mc, and neighboring follicle epithelial cells, we could selectively hone in on similarities and weed out genes expressed by DP but not preferentially relative to their neighbors. Interestingly, and unexpectedly, the DP signature was divergent from all cell categories to which parallels had been drawn previously.

Our DP signature contained most of the established DP markers. This was important, since no other published DP screen to date has provided a signature that accurately reflects the known DP expression program [11,12]. The failure of prior arrays to include most markers documented by in situ hybridizations and/or immunofluorescence is most likely attributable to the difficulties in purifying DP from the complex milieu of its surroundings and from the rapid loss of DP character that is known to happen when DP cells are taken out of their native niche and placed in culture. In addition, our Mx signature contained many of the established Mx markers and gave us the first glimpse at a global array profile enriched for this compartment of cells. Although the Mx itself is likely to be a mixture of early progenitor cells for all the differentiation lineages of the hair follicle, this total Mx profile will nevertheless be valuable in discerning how these cells diverge as they maintain their contact with the DP.

The ability to produce arrays that faithfully recapitulate the established programs of the DP and the Mx enabled us to capture new insights into the fascinating properties of these specialized cells and their potential for intercellular cross-talk. Amongst the most interesting features is the marked number of known hair disorder genes expressed by each of these cell types. Most of these genes, e.g., *Noggin* [37] and *Trps1* [26], were linked to hair diseases only within the past decade, concomitant with advances in positional cloning and mouse genetics. However, there are many more spontaneous and chemically induced mutations that have yet to be mapped and that involve hair phenotypes. Our arrays will be beneficial in accelerating the rate at which these diseases are mapped in the future.

As importantly as the contributions that these gene expression patterns make to establishing links between hair/skin genetic disorders and genes are the contributions that they make to our understanding of the underlying biology. Even for those cases where links between a disease gene and a hair disorder have been established, there is often little or no reliable data available for which of the cells of the hair follicle express the gene or how defects in the gene cause the morphological defects associated with the disease state. Examples in point are *Pthr1, Pdgfra, Tgfbr1, Egr2, androgen receptor,*and *Sox18,* which have all been implicated previously in hair disorders, but not recognized as genes that are preferentially expressed in a particular follicular compartment. Knowledge of the genes that are preferentially expressed by the epithelial and mesenchymal cells of the hair follicle provides a framework for functional studies to probe more deeply into the comprehensive biology involved. In this regard, disease genes are obvious candidates for governing the maintenance and character of a particular cell type, and their prevalence in our arrays provides perhaps the best evidence that these arrays are functionally significant.

The selective presence of known DP and hair disease genes in our DP signature gave us confidence in utilizing the list to better understand DP character and how it differs from dermal fibroblasts in stimulating the Mx cells of the hair follicle to differentiate. We were especially intrigued by a resemblance between the molecular differences in Mx and DP arrays versus those that are seen when neural and non-neural ectoderm segregate during embryonic neural induction [44]. During embryogenesis, when epidermal and neural lineages diverge, the epidermal lineages are determined by *Msx1*, *Dlx3/5*, and *Ap2* transcription factors, which subsequently control keratin gene expression, whereas the neural lineages are determined by *Sox2*, *Zic1*, and *Zic3* transcription factors, which subsequently lead to *Ncam1* and neural tubulin expression [44]. It is striking that in postnatal skin, the Mx signature of the hair follicle bulb is marked by the presence of *Msx1/2, Dlx3/Dlx2, Ap2,* and *keratin* genes, while the DP signature features *Sox2, Zic1, Zic3,* and *Ncam1*. In the future, it will be interesting to pursue the parallels between mesenchymal–epithelial cross-talk in the hair follicle and neuronal-epidermal cross-talk in embryonic development.

Our comparative analyses will also be valuable in the quest to realize the clinical potential for which the DP is known, namely for its inductive powers in hair growth. In this regard, we have uncovered a number of special features of the hair bulb microenvironment that provide tantalizing clues as to how DP cells exert their inductive powers. Among them are BMPs and BMP inhibitors, Shh inhibitory proteins, and Wnt signaling molecules. By comparing how the molecular signature changes when DP cells are removed from their niche and placed in culture, it should be possible to identify those genes whose expression is intrinsic to DP, and those whose expression is lost upon culture. Conversely, the constellation of molecular signature factors that are secreted by the native DP niche should then pave the avenue to employ a systematic approach to define the factors essential for maintenance of both the DP and the Mx signatures. A combinatorial effect will likely be necessary to fully unleash the full inductive potential of cultured DP, and knowing which surface receptors are expressed by DP and by Mx should expedite identification of these conditions.

In conclusion, our novel multicolor labeling approach demonstrates how array comparisons of highly purified cell populations can be employed to dissect the molecular make-up and cellular interactions within a complex microenvironment. Although we applied this strategy to unravel the molecular repertoire of closely interacting cell types within the hair follicle, the strategy is likely to be broadly applicable to a number of complex model systems.

## Materials and Methods

### Mice, cell isolation, FACS, and engraftments

For Lef1-RFP transgenic mice, a 6,713-basepair XbaI/NotI fragment of the human Lef1 promoter/5′UTR was cloned from a BAC (bacterial artificial chromosome) clone and assembled with RFP (DsRed-T1, a kind gift from B. Glick, University of Chicago, Illinois, United States). The K14-H2BGFP mice were previously generated in the lab [4]. Five backskins from P4 K14-H2BGFP/Lef1-RFP double transgenic mice were treated with dispase 4 °C, 8 h to separate epidermis/upper follicles from dermis. Dermis was digested with 0.2% collagenase at 37 °C, 40 min. Intact follicles and dermal cells were sedimented at 300 × g; follicles were obtained at 20 × g. Following trypsinization, 37 °C, 5 min, cell suspensions were strained. ORS and Mx cells were selected by FACS as GFP^high^RFP^−^ or GFP^low^RFP^−^ cells, respectively. DP were obtained after first depleting Mc (CD117^+^), lymphocytes (CD45^+^), and endothelial cells (CD34^+^) with biotinylated Abs (BD Pharmingen, San Diego, California, United States)/magnetic anti-biotin microbeads (Miltenyi Biotec, Bergish Gladbach, Germany), and then selecting for RFP^high^GFP^−^ cells in the FACS. The DF enriched in fibroblasts was the RFP^−^GFP^−^CD34^−^CD45^−^CD117^−^ population. For Mc isolation, cells were incubated with CD117-biotin followed by staining with streptavidin-APC (1:200, BD Pharmingen). Mc were purified by selecting RFP^high^CD117^+^ cells. Cells were stained, washed, and sorted in PBS/5% FCS. For dead cell exclusion, 300 ng/ml propidium iodide were added before FACS.

Cell isolations were performed on a FACSVantage SE system equipped with FACS DiVa software (BD Biosciences, Franklin Lakes, New Jersey, United States). Gates for fluorescence fractionation were set to match those approximated by semi-quantitative immunofluorescence analyses of the cell compartments. Cells were gated for single events and viability, then sorted. Cell purity was determined by postsort FACS analysis and typically was > 95%. For immunofluorescence characterization, cells were cytospun with a Cytospin4 unit (Thermo/Shandon, Pittsburgh, Pennsylvania, United States).

Engraftments were performed as described [7,9]. Experiments included a positive control of cell suspensions from freshly isolated WT dermis plus keratinocytes and a negative control of keratinocytes alone. Freshly isolated newborn keratinocytes (5–10 × 10^6^) and DP cells (2–4 × 10^6^) in first passage (1–2 wk of culture) were used for grafts. Hair typically appeared after 17–24 d.

### RNA isolation and microarray analyses

Total RNAs from FACS cells were purified using the Absolutely RNA Microprep kit (Stratagene, La Jolla, California, United States), and fluorometrically quantified (Ribogreen, Molecular Probes, Eugene, Oregon, United States). Quality was assessed by RNA 6000 Pico Assay (Agilent Technologies, Palo Alto, California, United States), and 800 ng were primed with oligo(dT)-T7 primer and reverse transcribed (Superscript III cDNA synthesis kit; Invitrogen, Carlsbad, California, United States). One round of amplification/labeling was performed to obtain biotinylated cRNA (MessageAmp aRNA kit; Ambion, Austin, Texas, United States), and 10 μg labeled cRNA was hybridized 45 °C, 16 h to mouse genome array MOE430a (Affymetrix, Santa Clara, California, United States). Processed chips were read by an argon-ion laser confocal scanner (Genomics Core Facility, Sloan Kettering Cancer Center, New York, New York, United States). Two entirely independent datasets were obtained for the five cell populations.

Scanned microarray images were imported into Gene Chip Operating Software (GCOS, Affymetrix) to generate signal values and absent/present calls for each probe-set using the MAS 5.0 statistical expression algorithm (.chp files). Each array was scaled to a target signal of 500 using all probe-sets and default analysis parameters. For comparisons, raw data and .chp files were imported into GeneTraffic 3.8 (Iobion Informatics, La Jolla, California, United States), and replicate microarrays were grouped and compared using the Robust Multi-Chip Analysis algorithm. Gene lists were compiled containing probe-sets > 2-fold increased for one over the four other populations. Probe-sets that scored as increased, but called absent were eliminated. Genes were grouped functionally by uploading probe-set lists to the “Database for Annotation, Visualization and Integrated Discovery” (DAVID 1.0) Web tool (http://apps1.niaid.nih.gov/david/upload.asp [46]).

### Semi-quantitative RT-PCR and real-time PCR

Total RNAs were purified from FACS-sorted cells as above, and after quantification with Ribogreen (Molecular Probes), normalized RNA quantities were reverse transcribed (Superscript III First-Strand Synthesis System, Invitrogen) using oligo(dT) primers. cDNAs were adjusted to equal levels by PCR amplification with primers to Gapdh. PCR amplification of genes of interest was performed using primers designed within the target sequence of the microarray probe-sets where possible, ensuring the uniqueness of the primers and the amplicon. All > 50 primer pairs were designed to work at the same settings: 3 min at 94 °C initial denaturing, 26–35 cycles of 15 s at 94 °C denaturing, 30 s at 60 °C annealing, and 25 s at 72 °C extension. For a list of primers used, see Table S14. Amplifications with minus reverse transcriptase control cDNAs yielded no products for any of the primer pairs at the cycles tested. For real-time PCR, the same primers were employed using the LightCycler System (Roche, Basel, Switzerland), LightCycler 3.5 software and the LightCycler DNA Master SYBR Green I reagents. Differences between samples and controls were calculated based on the 2^−ΔΔCP^ method.

### Cell culture

Viability of FACS-isolated DP cells was assessed by Trypan Blue (Sigma, St. Louis, Missouri, United States) staining, and equal numbers of live cells (5,000/cm^2^) were plated in Amniomax C-100 medium (Invitrogen), previously used for dog whisker DP cells [47]. This was the best of the five media tested.

### Immunofluorescence and in situ hybridizations

Lef1-RFP positive tissues were first fixed in a 37% formaldehyde buffer for 2–3 h and then embedded in OCT and frozen. All other tissues were immediately embedded in OCT, frozen, and sectioned. Paraformaldehyde-fixed sections and cytospin preparations were subjected to immunofluorescence or in situ hybridizations essentially as described [48,49]. When applicable, the MOM basic kit (Vector Laboratories, Burlingame, California, United States) was used to prevent non-specific binding of mouse monoclonal Abs. Abs and dilutions used were: AE13 (mouse, 1:50, [50]), AE15 (mouse, 1:50, [51]), Alx4 (mouse, 1:100, Exalpha, Maynard, Massachusetts, United States), BrdU (rat, 1:200, Abcam, Cambridge, Massachusetts, United States), CD104 (rat, 1:100, BD Pharmingen), Hhip (goat, 1:200, R&D Systems, Minneapolis, Minnesota, United States), Ki67 (rabbit, 1:500, Novocastra, Newcastle upon Tyne, United Kingdom), K5 (rabbit, 1:5,000, Fuchs Lab), K6 (rabbit, 1:1000, Fuchs Lab), Tyrosinase (rabbit, 1:500, kind gift from VJ Hearing), Vimentin (rabbit, 1:500, Biomeda, Foster City, California, United States), Wif1 (goat, 1:200, R&D Systems), HoxA9 (rabbit, 1:200, R&D Systems), p75 (rabbit, 1:100, Oncogene Science, Cambridge, Massachusetts, United States), Ncam1 (rat, 1:100, Chemicon International, Temecula, California, United States),. FITC, TexasRed, or Cy5 conjugated anti-mouse, -rat, -rabbit, or anti-goat Abs (1:200, Jackson Laboratories, Bar Harbor, Maine, United States) were used as secondary Abs. For detection of AP activity, the substrate 4-Nitroblue tetrazolium chloride/5-bromo-4-chloro-3-indolyl-phosphate 4-toluidine (NBT/BCIP, Roche) was used as recommended by the manufacturer's instructions. The following probes for in situ hybridizations were generated from IMAGE cDNA clones (IMAGE consortium, ATCC) using the DIG RNA labeling kit (SP6/T7) (Roche): *Bmp6* (Image:2779955), *Gfra1* (Image:6390018), *Hhip* (Image:6402422), *Mdk* (Image:4167496), and *Prss12* (Image:3665834). Nuclei were stained using 4-diamidino-2-phenylindole (DAPI). Imaging was performed using an Axioskop and Axiophot microscope (Carl Zeiss, Thornwood, New York, United States) equipped with Spot RT (Diagnostic Instruments, Sterling Heights, Michigan, United States) and Axiocam (Zeiss) digital cameras, respectively, or with an LSM 510 confocal microscope (Zeiss).

## Supporting Information

Figure S1 GFP High and Low Levels in Mature Anagen Phase Hair Follicles Two full-length hair follicles are shown, labeled with DAPI to mark the nuclei, anti-keratin 5 (red) and epifluorescence to show H2BGFP (green). Note that there is an approximately 3-fold greater level of GFP fluorescence in the ORS versus the Mx. Also note that every ORS cell along the length of the hair follicle co-expresses cytoplasmic K5 and nuclear H2BGFP, but not every ORS nucleus is present in each frozen section.
(5.8 MB TIF).

Figure S2 Minimal Contribution of Early Differentiating Cells to the Undifferentiated Mx Fraction To assess the contribution of terminally differentiating hair shaft cells to the undifferentiated Mx fraction, FACS-isolated cell populations were analyzed by immunofluorescence. Frozen skin sections (hair bulb) and cytospin populations were stained with Abs for AE13, AE15, and Keratin-6 (K6), which are expressed in the (pre-) cortex, IRS/medulla, and medulla/companion layer, respectively. DAPI (blue) and H2BGFP (green) are also shown in each immunofluorescence image. Cytospin quantifications (right) show that only 3–7% of the sorted Mx cells were positive for these differentiation markers. These cells most likely represent early differentiating cells that reside in the upper Mx area (arrows). Most of the other terminally differentiating cells of the hair follicle are eliminated in the trypsinization step, which is not sufficiently robust to dislodge the firmly adherent differentiating cells from the hair shaft.
(5.7 MB TIF).

Figure S3 Variable AP Activity of Sorted DP Cells and Functional Hair Reconstitution Assay (A and B) AP activity in FACS isolated DP cells. Cytospun sorted DP cells showed two levels of AP activity. While the majority of cells were strongly stained, a minor fraction showed weak reactivity ([B], arrows, bottom panel). Sorted ORS cells served as control ([B], top panel). Lower AP levels were detected in vivo in the most proximal part of DP (not shown).
(C) Functional hair reconstitution with FACS-isolated DP cells. Newborn keratinocytes were grafted onto backskins of *Nude* mice along with FACS-purified DP cells that were cultured 1–2 wk. After 3 wk, grafts were photographed. Note: it is well-established that dermal fibroblasts do not have this ability, which is unique to the DP cells and perhaps a few mesenchymal cells associated with the DP at the base of the follicle [7,9,19].
(3.5 MB TIF).

Figure S4 RT-PCR Confirmation of Molecular Backbone Genes Semi-quantitative RT-PCR of genes of the Molecular Backbone group of present and unchanged genes. Shown are representative examples of genes involved in RNA and protein metabolism.
(1.0 MB TIF).

Figure S5 Real-Time PCR Confirmation of Signature Genes and Correlation with Microarray Results Real-time PCR confirmation of selected novel and control genes from Figures 2C and 5A. Note the consistent distribution of genes between cell populations using both PCR methods (top panels and Figures 2C and 5A). As a measure of the performance of the microarrays, average signal values were plotted for each gene along with the real-time PCR results (bottom panels). Note the near-perfect match at the quantitative level.
(3.5 MB TIF).

Table S1 Correlation Coefficients of Array Hybridizations Raw data of correlation coefficients as shown in Figure 3A. The *p*-values of replicates are highlighted in bold.
(16 KB XLS).

Table S2 Microarray Expression Reports Compilation of quality control statistics for each of the ten microarrays (five fractions, two replicates) arranged in separate spreadsheets.
(104 KB XLS).

Table S3 Molecular Backbone Genes Complete list of Molecular Backbone Genes with average array signals and present/absent calls.
(1.5 MB XLS).

Table S4 Mx Signature Genes Complete list of Signature Genes with average array signals and present/absent calls, and average fold changes compared to each other fraction. Note that for convenience of access the genes are hyperlinked to the NCBI LocusLink/EntrezGene entries.
(130 KB XLS).

Table S5 ORS Signature Genes As in Table S4.
(4.4 MB XLS).

Table S6 DP Signature Genes As in Table S4.
(165 KB XLS).

Table S7 Mc Signature Genes As in Table S4.
(238 KB XLS).

Table S8 Significance Analysis of GO Categories of the Molecular Signatures and the Molecular Backbone Molecular Signature and Backbone genes were grouped functionally into GO categories and statistically analyzed with the “Database for Annotation, Visualization and Integrated Discovery” (DAVID 1.0) Web tool. The table contains the *p*-values of the Fisher exact probability test and the more conservative EASE Score as a statistical measure of enrichment of genes within GO categories. The values for each Molecular Signature and the Molecular Backbone group are arranged in separate tabs.
(742 KB XLS).

Table S9 GO Classification of Molecular Backbone Complete list of genes with corresponding GO classifications. Note that each gene may be in several categories. GO systems were split into separate tabs. Biological Process, BP; Molecular Function, MF; Cellular Component, CC.
(5.0 MB XLS).

Table S10 GO Classification of Mx Signature Complete list of genes with corresponding GO classifications. Note that each gene may be represented in several categories. The list is sorted for classifications, and each gene is hyperlinked to the NCBI LocusLink/EntrezGene entries.
(937 KB XLS).

Table S11 GO Classification of ORS Signature As in Table S10.
(1.81 MB XLS).

Table S12 GO Classification of DP Signature As in Table S10.
(1.3 MB XLS).

Table S13 GO Classification of Mc Signature As in Table S10.
(1.8 MB XLS).

Table S14 Primers for RT-PCR and Real-Time PCR Primer sequences for RT-PCR and real-time PCR. The same primers were employed for both RT-PCR methods.
(24 KB XLS).

### Accession Numbers

The Entrez Gene GeneID (http://www.ncbi.nlm.nih.gov/entrez/query.fcgi?db=gene) accession numbers for the genes and gene products discussed in this paper are: *a* (50518), *Akp2* (11647), *Bambi* (68010), *Bmpr1a* (12166), *Bnc* (12173), *Cldn1* (12737), *Cspg2* (13003), *Dlx2* (13392), *Dlx3* (13393), *Egr2* (13654), *Ets2* (23872), *Fgf10* (14165), *Fgf7* (14178), *Fgfr1* (14182), *Fgfr2* (14183), *Fn1* (14268), *Foxn1* (15218), *Foxq1* (15220), *Frzb* (20378), *Fst* (14313), *Gcl* (23885), *Gdf10* (14560), *Gfra1* (14585), *Gsdm* (57911), *Hhip* (15245), *Hoxc13* (15422), *Hr* (15460), *Inhba* (16323), *Itgb4* (192897), *Mki67* (17345), *Kit* (16590), *Krt1–14* (16664), *Krt1-c29* (16675), *Krt2–5* (110308), *Lef1* (16842), *Mdk* (17242), *Mlph* (171531), *Msx1* (17701), *Msx2* (17702), *Ncam1* (17967), *Nes* (18008), *Ngfr* (18053), *Nog* (18121), *Ovol1* (18426), *Pax3* (18505), *Pdgfra* (18595), *Prdc* (23893), *Prss12* (19142), *Ptch* (19206), *Pthr1* (19228), *Sca-1* (110454), *Serpine2* (20720), *Sfrp2* (20319), *Shh* (20423), *Snai2* (20583), *Snai1* (20613), *Sostdc1* (66042), *Sox10* (20665), *Sox18* (20672), *Sox2* (20674), *Sox9* (20682), *Tcf3* (21415), *Tcfcp2l2* (195733), *Tgfb1* (21803), *Tgfbr1* (21812), *Trps1* (83925), *Twist1* (22160), *Twist2* (13345), *Tyrp1* (22178), *Vdr* (22337), *Vim* (22352), *Wif1* (24117), *Wnt1* (22408), *Wnt10b* (22410), *Wnt3a* (22416), *Wnt5a* (22418), *Zic1* (22771), and *Zic3* (22773).

Raw data and normalized microarray expression data have been deposited at the Gene Expression Omnibus (GEO, http://www.ncbi.nlm.nih.gov/geo/) under the accession number GSE3142.

## Abbreviations

Abs: antibodies
AP: alkaline phosphatase
DF: dermal fraction
DP: dermal papilla
ECM: extracellular matrix
FACS: fluorescence activated cell sorting
GFP: green fluorescent protein
GO: Gene Ontology
IRS: inner root sheath
K14: keratin 14
K5: keratin 5
Mc: melanocyte
Mx: matrix
ORS: outer root sheath
P[number]: postnatal day [number]
RFP: red fluorescent protein
SC: stem cell
SKP: skin-derived precursor cell
